# Editorial

**DOI:** 10.5772/58757

**Published:** 2014-01-01

**Authors:** Markus Dettenhofer, Winston Patrick Kuo

**Affiliations:** 1 CEITEC Executive Director, Brno, Czech Republic; 2 Harvard Medical School, Boston, USA

It is a great pleasure to present the first volume of *Nanobiomedicine* (NBM). This unique open-access scientific journal will be available at no charge to both readers and authors, and aims to publish up-to-date expert reviews, scientific manuscripts, technology reports, perspectives, opinions and editorials in the rapidly emerging field of nanotechnology and biomedical research.

This feature will enable the clinical and scientific community to publish their latest ground-breaking research, up-to-date reviews, perspectives and opinions quickly and at the same token, readers around the globe will be able access them online as soon as they are published.

The journal will cover a broad range of topics, and at times selected themes, from translational and clinical applications that address the challenges and advances in diagnostics, to the delivery of therapeutics, to the monitoring of human diseases both in academia and industry. The fields that interface with nanotechnology and biomedical research include, but are not limited to, bioengineering, biophysics, biochemistry, physiology, pathology, nanotechnological applications in diagnostics, therapeutic application, vaccines, drug discovery/delivery and regenerative medicine.

As our first task, the Editors-in-Chief have and will actively expand our Editorial Board and develop collaborations/partnerships with international societies through the conferences, meetings and webinars we will be attending, including recruiting scientific experts who understand how discovery moves from lab to the market, and will look to attract contributors and participation from a more global audience. These experts will serve as the central hub of the nanotechnology and biomedical research community and provide us with guidance on the latest advances.

*Nanobiomedicine's* manuscript processing and peerreviewing is conducted entirely online. The Editorial Manager facilitates the manuscript processing time, thereby reducing costs and allowing for a better experience for our authors and reviewers. We have received numerous positive comments on this evolving electronic system, and we are indebted to our publisher, InTech, for managing this program for us. For more information, please visit our new manuscript submission system at http://www.intechopen.com/journals/show/nanobiomedicine/submit-your-paper. In addition, we are looking to expedite the acceptance to publishing time for our authors and will request informal opinions from our Editorial Board for borderline cases.

*Nanobiomedicine* will continue to work on partnering with other scientific societies, and academic and industry leaders to develop and advance the nanotechnology and biomedical research field and to allow free access to the readers.

We finally would like to thank InTech Open Access Publisher for supporting the initiatives of this journal and acknowledge *Nanobiomedicine's* Managing Editor, Ms. Petra Nenadic, and her team for their continuous support, passion and dedication all of which helped bring this project to fruition. In addition, we would like to thank our highly-renowned and respected Associate Editors and Editorial Board members from all over the world, whose areas of expertise range from nanofluidic systems, to nano-scale drug delivery, to nanoimaging, and who act as both bridges and catalysts to new research in their respective fields of nanotechnology and biomedical research.

